# Robot-assisted gait training yields superior functional gains in non-ambulatory subacute stroke patients

**DOI:** 10.3389/fresc.2026.1897418

**Published:** 2026-09-23

**Authors:** Alice Najsrova, Iva Fiedorova, István Szegedi, Sarka Banikova, Tomas Adamec, Adela Konde, Pavla Hanzlikova, Aravind Ganesh, Michal Bar, Ondrej Volny

**Affiliations:** 1 Department of Clinical Neurosciences, Faculty of Medicine, University of Ostrava, Ostrava, Czechia; 2 Department of Rehabilitation and Sports Medicine, University Hospital Ostrava, Ostrava, Czechia; 3 Department of Rehabilitation and Sports Medicine, Faculty of Medicine, University of Ostrava, Ostrava, Czechia; 4 Department of Neurology, Faculty of Medicine, University of Debrecen, Debrecen, Hungary; 5 Department of the Deputy Director for Science, Research and Education, University Hospital Ostrava, Ostrava, Czechia; 6 Department of Applied Mathematics, Faculty of Electrical Engineering and Computer Science, Vysoka Skola Banska - Technical University of Ostrava, Ostrava, Czechia; 7 Radiodiagnostics, University Hospital Ostrava, Ostrava, Czechia; 8 Departments of Clinical Neurosciences and Community Health Sciences, Hotchkiss Brain Institute and the O'Brien Institute for Public Health, University of Calgary Cumming School of Medicine, Calgary, AB, Canada; 9 Department of Neurology, University Hospital Ostrava, Ostrava, Czechia

**Keywords:** balance, functional ambulation category, Lokomat, robot-assisted gait training (RAGT), stroke, subacute rehabilitation, subgroup analysis

## Abstract

**Introduction:**

A randomized controlled trial (NCT04910217) demonstrated that the addition of robot-assisted gait training (RAGT) using the Lokomat Pro FreeD to protocol-defined conventional rehabilitation in the early subacute phase of first-ever ischemic stroke produced significant gains in functional ambulation category (FAC), Timed Up and Go (TUG), and Berg Balance Scale (BBS). Whether these benefits differ across clinically relevant subgroups remains unclear. We investigated potential effect modification by baseline characteristics.

**Methods:**

We performed a secondary analysis of a randomized controlled trial (*n* = 42 randomized; 36 completed follow-up: 17 RAGT, 19 conventional). Outcomes included ≥2-point FAC improvement and changes in BBS and TUG at 3 months. Subgroups were defined by stroke localization, baseline FAC (0–1 vs. 2–3), age, and recanalization treatment. Interaction effects were assessed using logistic regression for FAC; continuous outcomes were compared within subgroups using Mann–Whitney tests. No correction happened for multiple comparisons.

**Results:**

The largest differential effect of RAGT was observed in patients with low baseline ambulatory capacity (FAC 0–1): 90.0% of RAGT patients versus 45.5% in the conventional group achieved ≥2-point FAC improvement at 3 months (*p* = 0.039, *n* = 15). BBS gains were higher in this subgroup (median 27 vs. 12 points; *p* = 0.031), although this may partly reflect lower baseline values and greater recovery potential. Greater TUG improvement was observed in patients with anterior circulation stroke (*p* = 0.041). No subgroup showed a reversal of treatment effect.

**Conclusion:**

RAGT may confer greater benefit in patients with severe initial gait impairment. These exploratory findings support targeted evaluation in larger trials.

## 1 Introduction

Post-stroke gait impairment remains one of the most burdensome consequences of ischemic stroke, affecting up to 70% of survivors and profoundly limiting participation and quality of life (1). A substantial body of evidence, including Cochrane-level meta-analyses, confirms that the addition of robot-assisted gait training (RAGT) to conventional rehabilitation improves the likelihood of regaining independent ambulation after stroke, with larger effects observed in initially non-ambulatory patients. However, it remains unclear whether these differences reflect true effect modification or are driven by baseline functional status and recovery potential (2). The Lokomat Pro FreeD (Hocoma AG, Switzerland) enables highly controlled, repetitive, and progressively adapted gait movements delivered in the biologically critical early post-stroke window, thereby capitalizing on heightened neuroplastic responsiveness (3).

Our group recently published the primary results of a single-center randomized controlled trial (NCT04910217) assessing the additive effect of 15 sessions of protocol-defined RAGT over 3 weeks, initiated within a median of 13 days from first-ever ischemic stroke onset (4). The trial demonstrated significant between-group differences in the primary outcome of FAC improvement ≥2 points at 3 months (94.1% vs. 63.2%; *p* = 0.044), as well as in TUG (*p* = 0.034) and BBS (*p* = 0.049). These results are in accord with a recent meta-analysis by Baronchelli et al., which found consistent TUG improvements but less definitive BBS effects in patients undergoing Lokomat-based therapy (5). A clinically relevant feature of the Lokomat Pro FreeD platform employed in our trial is its integrated Augmented Performance Feedback system, which provides patients with a real-time avatar displayed on an interactive screen (6). This digital feature positions the Lokomat Pro FreeD not merely as a passive orthotic device, but as an integrated eHealth platform that merges robotic biomechanical assistance with digitally enhanced behavioral engagement.

Despite growing evidence at the group level, the clinical application of RAGT would be substantially advanced by identifying patient subgroups deriving the greatest - or least - benefit from its use. Patient heterogeneity in stroke rehabilitation is well recognized: baseline ambulatory function, stroke localization, age, and prior recanalization treatment all plausibly modify neuroplastic responsiveness and biomechanical capacity to engage with exoskeleton-based training (7, 8). Emerging meta-analytic data suggest that the benefit of RAGT on balance is modulated by treatment duration and patient selection, underscoring the need for stratified analyses (9). Moreover, recent European Stroke Organisation guidelines explicitly call for individualized, stratified approaches to intensity and modality selection in motor rehabilitation after stroke (3, 10). In parallel, there is growing recognition in eHealth and mHealth that digital engagement features - such as gamification, real-time biofeedback, and avatar-based interaction - may enhance adherence and engagement; however, their contribution to differential treatment effects across patient subgroups remains uncertain, particularly within complex interventions such as RAGT (11, 12). The avatar-mediated biofeedback inherent to the Lokomat Pro FreeD platform represents a practical embodiment of this principle within inpatient RAGT, raising the hypothesis that its motivational and neurofeedback properties may confer differential advantages in specific patient subgroups - particularly those with more severe motor impairment who are least able to engage with conventional task-specific training.

The present secondary analysis was pre-specified before database lock and aims to investigate whether the superiority of RAGT over conventional rehabilitation differs across clinically defined subgroups. Given the pilot nature of the parent trial, and consistent with recommendations for secondary analyses of small RCTs (13), we approached these analyses as hypothesis-generating rather than confirmatory, and report results without correction for multiple comparisons. This approach provides effect estimates and directional hypotheses for prospective, adequately powered trials.

## 2 Methods

### 2.1 Parent trial and ethical considerations

This secondary analysis uses the complete dataset from the parent RCT (NCT04910217), conducted at the University Hospital Ostrava between June 2020 and November 2023. The parent trial design, inclusion and exclusion criteria, intervention protocols, and primary outcome measures are described in detail in the primary publication (4). Briefly, hospitalized patients with first-ever ischemic stroke confirmed by MRI, early mRS 2–4, and early FAC 0–3 were randomized 1:1 to 15 sessions of RAGT (Lokomat Pro FreeD) in addition to protocol-defined conventional multidisciplinary rehabilitation (RAGT group), or to conventional rehabilitation alone. Outcomes were assessed at baseline, after 3 weeks, and at 3 months. The Lokomat Pro FreeD system includes an augmented performance feedback module providing real-time avatar-based visual biofeedback during treadmill walking (12, 14). The study was approved by the Ethics Committee of the University Hospital Ostrava (reference 395/2020). All participants provided written informed consent. This secondary analysis was conducted in accordance with the Declaration of Helsinki and did not require additional ethical approval, as it relies exclusively on pre-existing anonymized data from the approved parent study.

The parent trial was registered with an anticipated enrollment of 80 participants; actual enrollment reached 42 randomized participants (36 with complete 3-month follow-up). This shortfall relative to the registered target was attributable to the COVID-19–related disruption of in-hospital rehabilitation and to strict eligibility criteria (76.6% of screened patients were excluded), as detailed in the primary publication (4). The present secondary analysis uses this complete, fixed dataset.

### 2.2 Subgroup definitions

Subgroups were defined *a priori* based on clinically motivated hypotheses and availability of baseline data. Four stratification variables were selected: (i) stroke localization (anterior circulation vs. posterior/vertebrobasilar circulation), (ii) baseline FAC (low: 0–1 vs. higher: 2–3), (iii) age (below vs. at or above the sample median of 67 years; this decision was data-driven, with the intention to achieve groups of equal sizes), and (iv) stroke treatment approach (recanalization treatment [intravenous thrombolysis ± mechanical thrombectomy] vs. conservative management). Baseline FAC stratification was motivated by evidence suggesting that RAGT may be particularly advantageous for severely impaired, initially non-ambulatory patients (2). Stroke localization was included based on known differences in the anatomical substrates of gait control: posterior fossa and brainstem strokes frequently produce distinct balance and cerebellar deficits (15). Age was selected as a modifier of neuroplastic capacity, and recanalization treatment as a proxy for residual tissue salvage and early remodeling potential (8).

### 2.3 Rehabilitation

Rehabilitation was performed as described in the original publication (4), the detailed protocol is also shown in Supplementary Table 1. As mentioned above, Lokomat ProFreeD also offers additional stimulation through gamification and biofeedback elements. The device is located in front of a screen showing an avatar or another animation. The therapist chooses the mode selectively depending on the required effect and the segment of the lower limb that needs particular attention. Based on the therapist-selected mode, the presentation can be that of (for example) an avatar moving through an environment, the quality of the exercise can be visualized by the avatar's facial expression, or simply by presenting curves. All of this gives the patient feedback and stimulates her/his motivation to strive for the best performance and to continue with the exercise program.

### 2.4 Outcome measures

The following outcomes were examined within each subgroup: (i) the proportion of patients achieving FAC improvement of ≥2 points from baseline to 3 months (primary outcome of the parent RCT); (ii) absolute BBS improvement from baseline to 3 months (continuous secondary outcome); and (iii) absolute TUG improvement from baseline to 3 months (continuous secondary outcome, feasible only in patients with FAC ≥3 at baseline). FAC, BBS, and TUG were assessed by blinded evaluators as described in the parent publication.

### 2.5 Statistical analysis

Subgroup analyses for the binary FAC endpoint were performed using Fisher's exact test for between-group comparisons within each stratum. Risk differences (RD) with 95% confidence intervals (CI) were calculated using the Miettinen-Nurminen method. Treatment effect modification was assessed using logistic regression models including treatment, subgroup, and their interaction term. Continuous outcomes (BBS, TUG) were compared using the Mann–Whitney U test within strata; interaction effects were assessed using linear regression on rank-transformed data. All subgroup-level data were derived from the parent trial dataset using identical variable definitions and coding as in the primary publication (4). All analyses were conducted using R version 4.2.3. Given the exploratory nature of this analysis, a significance level of 0.05 was used as a nominal threshold without correction for multiplicity; all findings are interpreted as hypothesis-generating. Complete subgroup-level data, including individual patient-level values for all stratum variables, are available from the corresponding author of the parent trial on reasonable request.

## 3 Results

### 3.1 Population and subgroup distribution

Of 42 randomized patients, 36 completed the full 3-month follow-up and were included in the present analysis (17 RAGT, 19 conventional). The overall study population has been described in detail in the primary report (4). Baseline subgroup distributions were broadly balanced between groups (Table 1), although patients in the conventional group were older and had a higher burden of comorbidities, as reported in the original trial.

**Table 1:** Baseline subgroup distribution.

Characteristic	RAGT (*n* = 17)	Conventional (*n* = 19)	*p*
Age, years, median (IQR)	63 (55–68)	69 (64–71)	–
Stroke localization, *n* (%)			
Anterior circulation	12 (70.6)	14 (73.7)	1.000
Posterior circulation	5 (29.4)	5 (26.3)	1.000
Baseline FAC, *n* (%)			
0–1 (low)	10 (58.8)	11 (57.9)	1.000
2–3 (higher)	7 (41.2)	8 (42.1)	1.000
Hypertension, *n* (%)	13 (76.5)	19 (100.0)	0.040
Diabetes mellitus, *n* (%)	5 (29.4)	14 (73.7)	0.018
Metabolic syndrome, *n* (%)	8 (47.1)	12 (63.2)	0.503

Data are shown for the analysis population (patients with complete 3-month follow-up). Categorical variables are presented as *n* (%) and compared using Fisher's exact test; percentages are calculated from the group denominators (RAGT *n* = 17; conventional *n* = 19). Baseline characteristics of the full randomized cohort (*n* = 42) are reported in the primary publication.

IQR, interquartile range; FAC, functional ambulation category; RAGT, robot-assisted gait training.

– Continuous age was not re-tested in this secondary analysis; between-group age distribution in the parent cohort is reported in the primary publication.

### 3.2 Functional ambulation category: subgroup analysis

Results of the subgroup analysis for the primary FAC endpoint (≥2-point improvement at 3 months) are presented in Table 2 and Figure 1. In the overall sample, 94.1% of RAGT patients versus 63.2% of conventional patients achieved this endpoint (*p* = 0.044), consistent with the primary analysis.

**Table 2:** Subgroup analysis of FAC improvement (≥2 points at 3 months).

Subgroup	RAGT (*n* = 17)	Conventional (*n* = 19)	Within-subgroup *p*-value	Risk difference (95% CI)	*p*-interaction	Direction
Overall	94.1% (16/17)	63.2% (12/19)	0.044	+30.9 pp (+5.3 to +54.8)	–	Favors RAGT
Stroke localization						
Anterior circulation	91.7% (11/12)	57.1% (8/14)	0.083	+34.5 pp (+2.1 to +62.4)	0.61	Trend favors RAGT
Posterior circulation	100% (5/5)	80.0% (4/5)	>0.999	+20.0 pp (−17.3 to +59.7)	0.61	Both groups high
Baseline FAC						
Low (0–1)	90.0% (9/10)	45.5% (5/11)	0.039	**+44.5 pp (+2.4 to +74.2)**	**0**.038*	Favors RAGT
Higher (2–3)	100% (7/7)	87.5% (7/8)	>0.999	+12.5 pp (−14.6 to +44.9)	0.038*	Both groups high
Age						
<67 years	100% (9/9)	70.0% (7/10)	0.105	+30.0 pp (+2.5 to +56.8)	0.74	Trend favors RAGT
≥67 years	87.5% (7/8)	55.6% (5/9)	0.289	+31.9 pp (−9.1 to +64.7)	0.74	Trend favors RAGT
Treatment type						
Recanalization	100% (9/9)	77.8% (7/9)	0.211	+22.2 pp (−4.3 to +52.1)	0.83	Trend favors RAGT
Conservative	87.5% (7/8)	50.0% (5/10)	0.163	+37.5 pp (−2.8 to +68.9)	0.83	Trend favors RAGT

Within-subgroup *p*-values derived from Fisher's exact test. Risk differences calculated by Miettinen-Nurminen method. *p*-interaction from logistic regression including treatment, subgroup indicator, and multiplicative interaction term.

CI, confidence interval; FAC, functional ambulation category; pp, percentage points; RAGT, robot-assisted gait training.

*Nominally significant interaction *p*-value; interpret as exploratory directional signal only - individual strata are critically underpowered (*n* < 15 per stratum). Highlighted cells (green bold) indicate nominally significant results.

**Figure 1:**
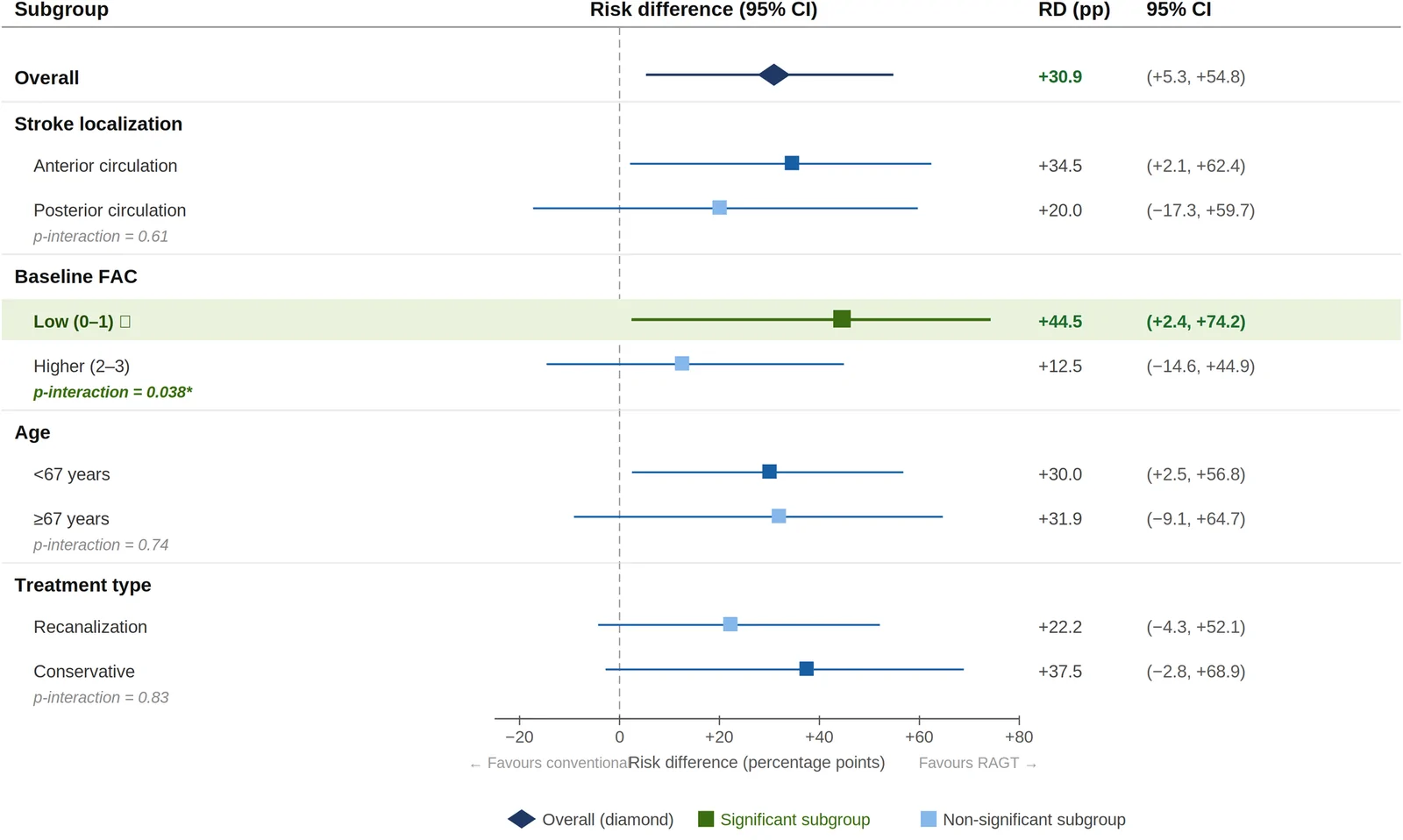
Forest plot of subgroup analyses: FAC improvement ≥2 points at 3 months. Forest plot showing risk differences (RD) with 95% confidence intervals (Miettinen-Nurminen method) for the primary endpoint (FAC improvement ≥2 points at 3 months) across pre-specified subgroups. Positive values indicate a treatment effect favouring robot-assisted gait training (RAGT) over conventional rehabilitation. The diamond represents the overall treatment effect. Filled squares represent subgroup-specific point estimates; horizontal lines represent 95% confidence intervals. The green highlighted row (★) denotes the primary subgroup of interest (baseline FAC 0−1). Square size does not encode sample size. CI, confidence interval; FAC, functional ambulation category; pp, percentage points; RAGT, robot-assisted gait training; RD, risk difference. *p*-interaction values derived from logistic regression models including treatment group, subgroup indicator, and their multiplicative interaction term. *p-interaction = 0.038 for the baseline FAC stratum: nominally crosses the pre-specified significance threshold but should be interpreted as an exploratory directional signal only, given critical underpowering of individual strata (*n* < 15 per stratum). No subgroup showed reversal of treatment effect (all RDs positive).

The most striking subgroup-specific effect was observed in patients with low baseline FAC (0–1): 90.0% of RAGT patients achieved ≥2-point FAC improvement, compared with only 45.5% in the conventional group (*p* = 0.039). The absolute risk difference was +44.5 percentage points (95% CI +2.4 to +74.2), indicating a clinically meaningful (although, given the small stratum size, imprecisely estimated) effect. The interaction *p*-value for the baseline FAC stratum by treatment interaction was 0.038; while this nominally crosses the pre-specified significance level, it should be regarded as a directional signal warranting prospective evaluation rather than confirmatory evidence of effect modification, given the critical underpowering of individual strata. In patients with higher baseline FAC (2–3), both groups performed well (100% vs. 87.5%; *p* > 0.999), and no meaningful differential benefit of RAGT was apparent.

For stroke localization, both anterior and posterior circulation strata showed directional trends favoring RAGT (91.7% vs. 57.1% in anterior; 100% vs. 80.0% in posterior), although neither reached statistical significance. The interaction *p*-value for localization by treatment was 0.61, indicating no statistically significant effect modification by stroke localization for the FAC endpoint.

Age-stratified analyses revealed consistent directional trends favoring RAGT in both the younger (<67 years) and older (≥67 years) subgroups, without statistically significant interaction (interaction *p* = 0.74). Recanalization treatment did not meaningfully modify the RAGT treatment effect on FAC (interaction *p* = 0.83).

### 3.3 Balance and mobility: BBS and TUG subgroup analysis

Subgroup data for BBS and TUG are summarized in Table 3. In the overall sample, median BBS improvement from baseline to 3 months was 28 points (IQR 19–36) in the RAGT group versus 15 points (IQR 10–28) in the conventional group (*p* = 0.049). In the low-baseline-FAC subgroup, BBS gains were notably larger in RAGT patients (median 27 points) compared with conventional patients (median 12 points; *p* = 0.031), suggesting a particularly pronounced effect in severely impaired patients. In the higher-baseline-FAC subgroup, a directional but non-significant trend also favored RAGT (30 vs. 20 points; *p* = 0.198).

**Table 3:** Subgroup analysis of balance and mobility outcomes (BBS and TUG improvement at 3 months).

Balance/mobility measure	RAGT	Conventional	*p*-value	Effect size (rank-biserial r)	*p*-interaction	Direction
A. Balance (BBS gain, points)						
Overall	28 (19–36)	15 (10–28)	0.049	*r* = 0.38 (moderate)	–	Favors RAGT
Baseline FAC						
Low (0–1)	27 (18–35)	12 (8–20)	0.031	*r* = 0.52 (large)	0.19^a^	Favors RAGT
Higher (2–3)	30 (22–38)	20 (12–31)	0.198	*r* = 0.33 (small-moderate)	0.19^a^	Trend favors RAGT
B. Mobility (TUG improvement, seconds)						
Overall (feasible pts)	11.2 (10.4–12.3)	6.4 (4.6–8.1)	0.034	*r* = 0.45 (moderate-large)	–	Favors RAGT
Stroke localization						
Anterior circulation	10.9 (9.6–12.1)	6.1 (4.2–7.8)	0.041	*r* = 0.49 (moderate-large)	n.s.	Favors RAGT
Posterior circulation	12.0 (*n* = 2)	7.2 (*n* = 4)	n/a	n/a (*n* < 3)	n/a	Descriptive only

Values presented as median (IQR). TUG assessable only in patients with baseline FAC ≥3. Posterior stroke TUG subgroup not interpretable due to *n* < 3 per cell.

Effect sizes reported as rank-biserial correlation (*r*): 0.1 = small, 0.3 = moderate, 0.5 = large (Cohen's conventions adapted for non-parametric tests). *p*-interaction from linear regression on rank-transformed data.

BBS, Berg Balance Scale; IQR, interquartile range; RAGT, robot-assisted gait training; TUG, Timed Up and Go test.

a Not reaching nominal significance; wide CIs due to critical underpowering of individual strata. n/a, not applicable.

TUG improvement was significantly greater in RAGT patients with anterior circulation strokes (10.9 s vs. 6.1 s; *p* = 0.041). In patients with posterior circulation strokes, TUG was assessable in only two RAGT patients and four conventional patients at baseline, precluding any meaningful subgroup comparison, and the interaction term for stroke localization by treatment for TUG was not significant.

## 4 Discussion

The present pre-specified secondary analysis of a single-center RCT provides the first subgroup-level evidence on the efficacy of RAGT in the very early subacute phase of first-ever ischemic stroke. Three principal findings emerge. First, the benefit of RAGT on functional ambulation (FAC ≥2-point gain at 3 months) is most pronounced in patients with the lowest baseline ambulatory function (FAC 0–1). Second, improvement in balance, as measured by the BBS, is also greatest in this severely impaired subgroup. Third, across all other clinically relevant subgroups, outcomes consistently favored RAGT without evidence of treatment reversal.

The concentration of RAGT benefit in the most severely impaired subgroup is conceptually coherent. Patients with FAC 0–1 have little or no independent stepping capacity, and are therefore unable to engage in meaningful overground practice within conventional rehabilitation. The Lokomat Pro FreeD, by providing controlled, graded body-weight support and progressive guiding force reduction, enables task-specific, high-repetition gait training even in patients who cannot achieve independent stepping with conventional methods (4). This mechanistic explanation aligns with the Cochrane meta-analysis by Mehrholz et al., which found that the advantages of electromechanical-assisted gait training were most pronounced in initially non-ambulatory patients (2). It is also consistent with the meta-analytic data by Loro et al., which observed greater BBS gains from RAGT in more severely impaired patients (9).

The Lokomat system incorporates real-time visual biofeedback and goal-directed tasks that may enhance engagement and motor learning; however, their contribution to differential treatment effects across subgroups cannot be determined from the present data (12, 14). The integration of such feedback-driven and interactive elements is consistent with broader eHealth and mHealth principles, where digital engagement features have been shown to improve adherence and rehabilitation outcomes in neurological populations (12). These effects may be particularly relevant in severely impaired patients (FAC 0–1), who have limited capacity for independent task-specific practice and may benefit disproportionately from externally guided, feedback-enhanced training. Future studies should evaluate whether device-derived engagement metrics mediate or moderate treatment effects across patient subgroups.

The differential TUG benefit in anterior versus posterior circulation stroke deserves interpretive caution. Posterior fossa strokes frequently produce cerebellar ataxia and vestibular dysfunction, which may limit the therapeutic relevance of exoskeleton-guided stepping patterns that primarily train lower-limb kinematics (15). Conversely, anterior circulation strokes predominantly cause hemiparetic gait, which the Lokomat's bilateral orthotic guidance is mechanistically well-suited to address. The significant TUG finding in anterior-stroke patients (*p* = 0.041) is, therefore, plausible, but the near-zero sample size in the posterior-stroke TUG subgroup (*n* = 2 RAGT, *n* = 4 conventional) limits any definitive conclusion, and interaction analyses did not reach significance.

Age did not appear to modify the treatment effect in this sample. This finding contrasts with some meta-analytic evidence suggesting attenuated neuroplastic responsiveness in older patients (7), but the relatively narrow age distribution in our sample (IQR 58–71 years overall) may have precluded the detection of age-related modification. Importantly, the absence of a statistically significant age-by-treatment interaction, combined with directional trends favoring RAGT in both age strata, supports the applicability of RAGT across the age range represented in our trial. Similarly, recanalization treatment did not modify the treatment effect on FAC, suggesting that the benefit of adding RAGT is not contingent on prior thrombolytic or thrombectomy-mediated reperfusion.

Several methodological considerations warrant explicit acknowledgment. This analysis, while pre-specified, was conducted on a pilot RCT with a total analyzed sample of 36 patients, and subgroup cells range from 5 to 14 patients per arm. As a consequence, all subgroup-specific comparisons are critically underpowered, and even the nominally significant findings (baseline FAC 0–1 for both FAC and BBS; anterior stroke for TUG) carry wide confidence intervals that preclude precise effect estimation. We have avoided reporting odds ratios in this context, as these are known to be inflated in sparse tables and to produce uninformative confidence intervals; risk differences with Miettinen-Nurminen CIs are reported instead, but these remain wide given the small stratum sizes. The interaction *p*-values reported in this analysis should be interpreted with caution: the statistical power to detect genuine treatment effect heterogeneity with *n* < 15 per stratum is well below 20%, meaning that non-significant interaction *p*-values cannot exclude meaningful differential effects, and nominally significant ones cannot confirm them. All subgroup-level data were derived from the parent trial dataset using identical variable definitions as in the primary publication.

The baseline imbalance between groups in comorbidity burden is an additional limitation: the conventional group had higher rates of hypertension (100.0% vs. 76.5%; *p* = 0.040) and diabetes mellitus (73.7% vs. 29.4%; *p* = 0.018). These imbalances, arising by chance in a relatively small RCT, may have confounded subgroup analyses that relied on individual patient-level covariates. No formal multivariable adjustment for baseline comorbidities was applied in the subgroup models due to the limited sample size; readers should interpret the reported effects with this caveat in mind.

Despite these limitations, the present study makes a meaningful contribution. To our knowledge, this is the first published subgroup analysis from a prospective RCT of RAGT that specifically addresses the very early subacute window (median 13 days from stroke onset), uses the Lokomat Pro FreeD with a standardized three-step progressive protocol, and reports balance outcomes alongside functional ambulation across clinically motivated strata. The finding that patients with FAC 0–1 at baseline derive the greatest benefit has direct implications for patient selection and prioritization of RAGT resources in clinical settings where device availability is limited.

Future research should prioritize adequately powered, multicenter RCTs designed *a priori* to investigate treatment effect modification in RAGT, with pre-registration of subgroup analysis plans. Based on the current findings, baseline ambulatory function (FAC 0–1 vs. 2–3) and stroke localization should be incorporated as stratification variables in future trials. Furthermore, the digital architecture of modern RAGT platforms, such as the Lokomat Pro FreeD (encompassing real-time avatar biofeedback, gamification elements, and automated session-level data recording), provides an opportunity to integrate RAGT into broader eHealth and mHealth frameworks for neurological rehabilitation. Specifically, automated data streams generated by the device (step count, guiding force reduction, training speed, session duration, avatar task performance) could be leveraged for remote monitoring, adaptive protocol adjustment, and individualized rehabilitation analytics - areas of active development in digital neurology (14). In the context of post-discharge care, integration of Lokomat-derived biofeedback data with mHealth platforms may enable continuity of avatar-guided exercise feedback beyond the inpatient setting, supporting engagement with home-based programs in the late subacute and chronic phases. Further research is also needed to elucidate the neurophysiological mechanisms - including cortical reorganization, cerebellar adaptation, and proprioceptive reweighting - that underpin differential balance and gait outcomes across patient strata, and to determine the extent to which gamification-enhanced engagement modifies these mechanisms.

The principal limitation of this trial, which arose despite block randomization based on FAC scores, lies in the imbalance between the study groups in hypertension and diabetes, both in favor of the RAGT group. As both these comorbidities may influence therapy success, it needs to be stated that the entire difference in rehabilitation results cannot be reliably attributed solely to the RAGT, and the results of our study should rather be considered indicative than a definite proof of RAGT treatment success.

## 5 Conclusions

This pre-specified secondary analysis of a single-center RCT provides evidence that RAGT using the Lokomat Pro FreeD produces its greatest additive benefits over conventional rehabilitation in patients with the most severe baseline ambulatory (FAC 0–1). Across other clinically meaningful subgroups, outcomes consistently favored RAGT without evidence of treatment reversal. These findings support further investigation of early RAGT, particularly in severely impaired patients, and provide hypothesis-generating effect estimates for future adequately powered trials.

## Data Availability

The raw data supporting the conclusions of this article will be made available by the authors, without undue reservation.
