# Supplementary material for: Robot-assisted gait training yields superior functional gains in non-ambulatory subacute stroke patients
Source: Front Rehabil Sci. 2026 Sep 23;7:1897418. doi: 10.3389/fresc.2026.1897418 (PMC13642629; doi:10.3389/fresc.2026.1897418)
Supplement: Supplementary file 1 [file Datasheet1.pdf]

Supplementary Table 1. Robot-assisted gait training protocol using the Lokomat device.

Timing	Training requirements per therapy session
**Week 1**	20–30 min; guidance force 80–100%; ≥1000 steps required; BWS at least 50%; stimulation: physiological gait pattern, coordination
**Week 2**	30–45 min; guidance force 60–100%; ≥1800 steps required; BWS 30–45%; stimulation: physiological gait pattern, strengthening of the phasic and stance (weight-bearing) function of the lower limbs, coordination
**Week 3**	35–50 min; guidance force 40–100%; ≥2200 steps required; BWS 20–45%; physiological gait pattern; stimulation: strengthening of the stance and phasic function, acceleration components and coordination, endurance

Legend

Guidance force — computer-controlled drive of the orthoses, allowing partial unloading or assistance; it determines the extent to which lower-limb movement is guided by the Lokomat on the treadmill according to the physiological gait pattern. At 100% guidance force, the orthoses are fully driven by the Lokomat and the patient moves according to a pre-set gait pattern.

Body weight support (BWS) — the system provides precise and dynamic unloading of part of the body weight during physiological gait training; the BWS value expresses the degree of unloading in kilograms, while the software displays the corresponding percentage of body weight.

Physiological gait pattern — coordinated alternation of the phasic and stance function of the lower limbs; throughout therapy, the therapist adjusts gait via the software to obtain the highest-quality afferent input, optimally with the patient's active participation.

Stimulation — goal-directed physiological gait; exercises with augmented performance feedback (training programs: step symmetry, acceleration training, strengthening of physical function, relaxation). Control elements are configured according to the targeted domain: e.g., hip flexion, by which the patient controls the game through this movement. The mix of the following games was used, depending on the therapist's experience and the required exercise: Body weight support and Speed; Speed; Virtual Walking; Stay on Course; Smile, Body Weight Support; Thirsty Tourist; Nature Island; ICE Hopper – Summit.

Therapy goals / therapy focus — movement pattern, endurance, attention and motivation, symmetry, timing of activity, general parameters.

Abbreviations: BWS, body weight support; min, minutes.

The program was in accordance with the recommendations by Akıncı et al.

Reference:

Akıncı, M., et al., 2025. Robot-Assisted Gait Training in Stroke Rehabilitation: A Multidisciplinary Overview on Lokomat Features. Istanbul Gelisim University Journal of Health Sciences, 27, 1145-1155. https://doi.org/10.38079/igusabder.1644772
